# The Environment–Economy Synergistic Improvement Effect of the “Two-Oriented Society” Pilot Area in China

**DOI:** 10.3390/ijerph20010852

**Published:** 2023-01-02

**Authors:** Chunying Cui, Dengke He, Ziwei Yan

**Affiliations:** 1 School of Economics and Management, Yiwu Industrial & Commercial College, Yiwu 322000, China; 2 Research Center of Internet and Industrial Development, Wenhua College, Wuhan 430074, China; 3 School of Economics and Management, Yangtze University, Jingzhou 434023, China

**Keywords:** environment–economy synergistic improvement, “two-oriented society” pilot area, difference-in-differences, synthetic control method

## Abstract

The establishment of the “two-oriented society” pilot zone is China’s effort to explore an economic–environmental synergistic growth approach, and it is an important basis on which to solve the dilemma between economic development and environmental protection in less developed countries. By constructing an inter-provincial panel dataset and taking the “two-oriented society” pilot area as a policy intervention event, a quasi-natural experiment was conducted to evaluate the observed differences in economic growth and pollutant emissions using counterfactual estimation. The results show that, during the policy intervention period, the emission of solid waste in Hubei and Hunan provinces was significantly reduced, and the level of haze particles in Hunan province was also remarkably suppressed; however, the environmental emission problems such as water pollution were not improved in comparison to the national level. At the same time, the economic growth rate of Hubei and Hunan provinces was clearly better than the counterfactual control group after the policy pilot, showing the economic promotion effect of the construction of the “two-oriented society” pilot zone. We conclude that the establishment of the “two-oriented society” provides a reference for a successful path to sustainable growth, and there is no absolute contradiction between economic growth and environmental friendliness.

## 1. Introduction

The proposed environmental Kuznets curve (EKC) reveals an inverted U-shaped relationship between economic development and the environmental scale. A range of experiences in developing countries demonstrates that there is a strong correlation between economic growth and environmental pollution and a trend of change in the same direction. Extensive empirical studies have evaluated a variety of environmental degradation indicators, including atmospheric pollutant emissions, carbon dioxide emissions, sulfur dioxide emissions, nitrogen oxide emissions, air particulate matter PM2.5 emissions, water pollution, soil pollution, and solid pollutant emissions [1,2]. According to research on samples of different economies in developed and developing countries, it has been found that different levels of economic development are associated with different stages of the EKC curve [3]. The majority of developing countries often fall into the first stage of the EKC curve. Faced with the correlation between economic growth and environmental degradation [4], it is difficult to achieve a trade-off between income and pollution emissions.

In recent years, the concept of sustainable development has gradually achieved a consensus among policymakers and the public. Economic practices in many developing countries are actively exploring how to deal with the coexistence between the environment and development. A large number of country-wide studies in Asian countries found that many economies did not display a causal link between economic growth and environmental degradation [5]. The governments of developing countries such as India and Turkey are striving to promote the use of clean energy while growing their economies and adopting environmental legislation to deal with pollution [6,7], which results in the classical EKC curve framework not being applicable to the transformation of environmentally inclusive societies.

With the continuous development of economic globalization, the majority of developing countries solve the problems of poverty and development by actively participating in world economic and trade activities, thus falling into the dilemma of increasing the national income while also increasing the pollution burden. Increasingly severe environmental and climate challenges threaten populations’ safety and health, as well as affecting the sustainability of economic and social growth [8,9]. Due to a lack of finance, technology, and effective institutional arrangements to deal with environmental problems [10,11], developing countries’ intentions are usually strong, but their actions are comparatively weak. Therefore, LMICs cannot sacrifice economic growth to meet the single environmental goal [12,13], and economic support must be provided for environmental governance through sustainable growth [14], so as to achieve the long-term, sustainable improvement of the environment and achieve the requirements of the UN Millennium Development Goals.

Many researchers have paid attention to China’s environmental pollution indicators, especially the reduction of the “three wastes” in the form of gas, liquid, and solid [15,16,17]. In such studies, the empirical analysis of air pollutants as indicators is extensive [18], including carbon dioxide emissions, atmospheric particulate PM2.5, sulfur dioxide and nitrogen oxide emissions, and other types of pollutants. Their emissions are all associated with economic growth [19,20,21]. However, recent empirical evidence demonstrates that pollutant emission levels have improved considerably [22,23,24,25].

Scholars have pointed out that, in China, there are many factors leading to the excessive emissions of the above pollutants, including economic growth and the energy structure relying too much on non-renewable energy [26], trade liberalization and large export surpluses resulting in increased emissions of pollutants [27,28,29,30], ECER technology progress and promotion [26], and urbanization and financial development [31,32,33,34].

As an important member of the group of developing countries, China is also actively exploring the coexistence between economic growth and environmental protection. Since the reform and opening up of the economy, China’s economy has been experiencing a period of rapid growth for more than 40 years, and the environmental consequences are becoming increasingly obvious. It is urgent to realize the coordinated development of economic growth and environmental improvement through institutional innovation.

Recently, academic studies on the “two-oriented society” pilot zone have emerged, which proves that the establishment of the “two-oriented society” pilot zone can achieve a win–win situation of economic growth, energy conservation, and emission reduction from many aspects, such as pollution levels and carbon emission intensity [35,36,37]. However, the existing research on energy conservation and emission reduction (ECER), limited to observing the emissions of sulfur dioxide and carbon dioxide, has failed to carry out a systematic analysis of environmental pollution control; alternatively, it may only focus on the change level of green productivity in a single province, without conducting an overall study of the pilot region, thus lacking generality. Therefore, this paper attempts to use counterfactual estimation and regard the establishment of the “two-oriented society” pilot zone as a quasi-natural experiment. We used the panel data of Chinese provinces from 2001 to 2016, and the pilot provinces of the “two-oriented society” pilot zone were taken as the treatment group, with other provinces as the control group. The synthetic control method was used to identify whether the construction of the “two-oriented society” pilot area has achieved a positive promotion effect in economic growth and environmental pollution control and has introduced an unusual means of green development.

Compared with previous studies, the possible new contributions of this work are as follows. (1) It is different from previous research using only a single test sample or a single emission reduction result, as this study focuses on the establishment of the “two type society” test area in Hubei and Hunan provinces to carry out a comprehensive evaluation, including the gas, liquid, and solid pollutant emission reduction conditions; it also finds that the regional economic growth was better in the same period than in other pilot provinces, showing the global relevance of the research. (2) The study reveals the coordinated development of the “two-oriented society” in the dimensions of energy conservation, emission reduction, and economic growth, reflecting a new, inclusive growth path beyond the environmental “Kuznets curve”.

## 2. Institutional Background and Research Hypothesis

### 2.1. Institutional Background

In December 2007, the State Council approved the overall plans of the National Comprehensive Reform supporting the pilot zone and selected Wuhan and Changsha as two urban agglomerations for the pilot, aiming to explore the outcomes of building an environmentally friendly and resource-conserving society (hereinafter referred to as “two-oriented society”). The Chinese central government has also adopted environmental legislation, involving an accountability system of officials and the establishment of environmental governance tournaments to actively innovate at the institutional level and strive to achieve high-quality development. Among them, the “National Comprehensive Reform Supporting Pilot Zone” system with Chinese characteristics has been piloted in Hubei and Hunan provinces with the theme of building an “environmentally friendly and resource-conserving society”.

The policy of the “two-oriented society” was enhanced by the attention level of the central government. Thus, many feasible policy measures were implemented in Hubei and Hunan, as shown below.

At first, the pressure to implement policies comes from the high-level decisions of the regime, and these measures were designed according to the targets of the environmental and growth objectives in reports of province work. The fifth Plenary Session of the 16th CPC Central Committee listed the construction of the “two-oriented society” as the strategic goal of economic and social development for the first time. The 17th National Congress of the Communist Party of China highlighted the strategic positioning of the construction of the “two-oriented society” pilot area, and the comprehensive reform of the Wuhan and Changsha city clusters supporting the pilot work has been widely regarded by society. The Wuhan Metropolitan Circle is an “8 + 1” cobweb urban agglomeration with Wuhan as the center city, accounting for 50% of the population and more than 60% of the GDP in Hubei province. The Changsha urban agglomeration refers to Changsha and its neighboring Zhuzhou and Xiangtan prefecture-level cities. It is a triangular urban circle of “Changsha–Zhuzhou–Xiangtan”, formed through long-term urban integration development, accounting for more than 20% of the population and 40% of the GDP of Hunan province. The development of the “two-oriented society” pilot zones in these two urban agglomerations determines the level of inclusive development of economic growth and environmental friendliness in Hubei and Hunan provinces.

Secondly, Hubei and Hunan provinces issued their own “two-oriented society” pilot zone “program” in 2008 and 2009. The “program” is related to economic growth, ECER policy documents, and a release system of dividends, and it has different degrees of effect on economic growth and ECER in Hubei and Hunan provinces. After the promulgation of the “program”, with the release of institutional dividends, mechanism reform has been continuously promoted, which has exerted an impact on ECER and economic growth. On the one hand, it controls air quality, energy consumption per unit output value, pollutant emissions, and carbon emissions; moves away from high-emission and energy-consuming enterprises; and introduces enterprises with energy conservation and environmental protection advantages to implement market access, which drives environmental improvement and, thus, plays a role in energy conservation and emission reduction. On the other hand, through industrial promotion, pilot policies achieve economic growth by promoting the development of advanced manufacturing, traditional industries with superior advantages, modern agriculture, producer services, foreign trade, high-tech industries, new energies, and new material industries.

The “program” has a significantly positive effect on ECER. In 2008, Hubei province proposed that energy consumption and sulfur dioxide emissions per unit of GDP should be significantly reduced. The market access mechanism of new projects needs to be further improved, and the market access threshold of emission reduction should not be lowered. Monitoring and controlling energy consumption and total pollution emissions to reduce waste generation will require active measures to control industrial pollution sources, encourage and advocate for cleaner production, and reduce the total volume of waste emission. The recycling and utilization of renewable resources has been further improved in order to better promote the development of industries related to renewable resources; implement the collection of municipal sewage and solid waste treatment fees; and strictly control the air quality, so that it meets the national secondary standards. Under the condition of strict law enforcement, environmental legislation can play a role in improving the environment [38].

In 2009, Hunan province stated that the energy consumption per unit of regional GDP and the emissions of major pollutants should not be higher than the national average. To carry out the pilot trade of emission rights, so as to promote it, it is necessary to restrict and close down some enterprises with high energy consumption and emissions and launch pilot projects to compensate for the withdrawal of industries; to further explore industrial waste treatment certification systems, so that ECER supervision and management can be effectively implemented; to support and develop a number of large enterprise groups with advanced energy conservation and environmental protection technologies and a strong driving force; to explore the establishment of extended producer responsibility and industrial waste treatment certification systems; and to strictly control the city’s air quality to meet the standards. In terms of the effect of emission reduction, China’s emission trading policy is effective [39].

Thirdly, in order to boost economic growth, local governments in the “two-oriented Society” pilot zone have developed environmentally inclusive economic revitalization plans. In 2008, Hubei province proposed to promote the optimization and upgrading of the industrial structure and achieve better development by changing the methods of economic development. For example, it proposes to make good use of local resources, advocate for energy and material savings, and actively promote green product standards; pool resources to develop advanced manufacturing and encourage and support traditional industries with advantages; attach importance to the development of modern agriculture to optimize and upgrade traditional agriculture; and focus on the construction of a circular economy industrial park and ecological industrial park, which should be determined by local resource endowment and industrial characteristics. The development of producer services should not be ignored, and the coordinated development of the three industries should be promoted, so as to encourage and support the development of foreign trade and promote foreign trade facilitation and focus on attracting some high-tech and high-value-added enterprises and research and development institutions. After the implementation of the industrial policy, this has a significantly positive promotional effect on the rationalization and upgrading of the industrial structure [40].

Hunan province proposed, in 2009, that the industrial layout should be reasonable and the regional division of labor should be clear. We should focus on advanced manufacturing and high-tech industries and actively provide support and assistance for their better development. We should further improve the mechanisms for the application of scientific and technological advances and increase efforts to develop high-tech industries such as new materials and new energy; encourage and support the development of industries that conserve resources and protect the environment, so as to change the growth pattern and consumption pattern; pay attention to talent development and allocation, to better promote all kinds of talent exchange and cooperation; and increase export processing zones based on the actual situation to develop foreign trade. After the establishment of the “two-oriented society” pilot zone, it promoted the GDP growth of Hunan province and improved the ecological efficiency of Hunan province [41].

Emphasizing that policy advantages are reflected in economic growth, it is necessary to verify whether the economic growth of the pilot area is better than that of other non-pilot provinces or whether it is necessary to sacrifice growth to protect the environment. In addition, the reduction effect of environmental emissions is better than that of other regions. Otherwise, only growth is taken into account while ignoring the environment. Therefore, high-quality growth combined with environmental constraints is the greatest institutional innovation challenge brought by the pilot zone of the “two-oriented society”.

### 2.2. Research Hypothesis

Previous research shows that structural factors of economic and social development affect the process of technological progress, thus restricting pollutant emission reduction. Although economic development leads to an increase in pollutant emissions, the growth of carbon emissions is influenced by the energy structure and energy intensity [42]. The optimization of the industrial structure can gradually drive the reduction of the regional pollutant emission level [43]. This structural change means that the upgrading of the industrial structure is a driving mechanism for the diffusion of ECER technology [44,45]. Evidence from world trade shows that openness to trade and investment contributes to increased pollution levels [46,47], and the import diffusion effect of advanced environmental protection technology and processing technology gradually inhibited the generation of the corresponding pollutants [48]. The re-understanding of the financial sector in economic development also reveals that the rise of green finance promotes the expansion of the energy-saving and environmental protection features of economic development [49] and promotes the rapid exit of existing high-carbon emission industries and the vigorous popularization of clean energy [50,51]. Therefore, structural factors play an important role in the effective reduction of carbon emissions and are the main mechanism and guarantee for the popularization of emission reduction technology.

Moreover, we cannot ignore the positive role of government environmental policies in the process of growth–environmental nexus decoupling. Studies illustrate that the reduction of pollutant emissions not only depends on the technical path, but it more strongly depends on the policy path [52]. In terms of environmental policies, policymakers’ actions regarding environmental improvement have many effects [53]. In addition to the effective establishment of environmental legislation and emission standards, strict compliance checks are also key indicators for the evaluation of policy implementation [54,55]. Simultaneously, in order to encourage the progress and diffusion of emission reduction technologies, a good intellectual property protection system is essential [56]. The establishment of a series of property rights trading markets in China has effectively solved the efficiency problem of the transfer of emission rights, and market mechanisms can be brought into play to achieve energy conservation and emission reduction [57,58]. It is worth noting that studies from the field of law and economics show that environmental legislation does not significantly reduce pollution emissions. Environmental legislation can only help to reduce pollution emissions in provinces with stronger enforcement [59]. Moderately strengthening environmental regulations can achieve a win–win situation of economic performance and environmental performance for groups with medium ecological efficiency [60]. Therefore, environmental accountability and other incentive methods have been widely adopted by the Chinese government and have formed long-term environmental governance effects [61,62].

In addition to the government’s environmental policies, another important means of environmental policy governance that have been ignored by the existing research is China’s special institutional zones. China tends to pilot institutional innovation by setting up pilot zones, and after gaining experience and lessons, the improved policies are used for nationwide popularization [63]. Since the economic reform, China has continuously carried out a series of special zone experiments, including trade liberalization, financial reform, urban–rural integration, offshore tourism, and other special zones, which have achieved excellent results [64,65]. The “two-oriented society” pilot zone is an institutional innovation pilot launched by the central government in Hubei and Hunan provinces. Therefore, it can be regarded as a special environmental policy intervention zone. In the two pilot regions, environmental friendliness and resource conservation are seen as key goals of local government governance, while policymakers are also required to maintain economic vitality without sacrificing development opportunities for environmental improvement or overextending environmental capacity for the speed of development. Some empirical conclusions have partially verified that the establishment of the “two-oriented society” pilot zone can reduce pollutant emissions in the pilot area [35,37]; carbon emission intensity and air pollution are also effectively controlled [66]. However, the evaluation of the overall environmental improvement of the pilot policy is still insufficient; in particular, the pollution abatement effect of local air, water, and soil has not been covered.

Finally, economic growth has been one of the main aspirations of China in setting up special zones and piloting advanced systems. A large number of research results on China’s policy pilot regions show that the policy dividend of the establishment of special economic zones will promote local economic growth without exception [67,68]. China’s reform policy tends to be pilot before promotion, and pilot areas of reform usually result in a higher economic development speed [69]. Recently, research on the policy of special zones to promote economic growth has mostly focused on the free trade area. Counterfactual studies on the free trade zones in Shanghai and Fujian demonstrate that such special zone policies have a great impact on the real per capita GDP [70,71]. A synthetic control method (SCM) study in the Manaus Free Trade Zone in Brazil also confirmed that the establishment of the free trade zone significantly increased the real GDP per capita and total output value of services per capita [36]. If the special zone is set up with the aim of environmental friendliness and resource conservation, it will not be sustainable if it does not present outstanding results in terms of economic growth. A recent study has adopted green total factor productivity (GTFP) calculation and confirmed that the pilot zone in Wuhan has driven urban green development, but further studies have not followed up on this [72]. No research has definitively demonstrated whether there is an economic boost at the provincial level.

Based on the above research results, there have been many studies on the reform pilot zone in recent years, most of which are limited to economic growth and do not explain whether high-quality development can be achieved. In addition, in the aspect of environmental governance research, there are abundant achievements, but most of them focus on the evaluation of the effect of environmental policy, limited to the policy intervention effect of technological progress and environmental legislation, without answering the question of whether the reform pilot zone can achieve the effect of ECER. Therefore, this paper describes a package counterfactual analysis of economic growth and solid, gas, and liquid emissions, in Hubei province and Hunan province, respectively, and confirms the policy effects of comprehensive reform pilot zones in terms of the economic growth rate and environmental pollution control.

Based on the above mechanism analysis, this paper proposes the following hypotheses:

**Hypothesis** **1a.** *The “two-oriented society” pilot area has a significant reduction effect in air pollution control.*

**Hypothesis** **1b.** *The “two-oriented society” pilot area has a significant reduction effect in water pollution control.*

**Hypothesis** **1c.** *The “two-oriented society” pilot area has a significant reduction effect in the treatment of solid pollutants.*

**Hypothesis** **2.** *The “two-oriented society” pilot zone promotes economic growth in Hubei province and Hunan province.*

## 3. Materials and Methods

### 3.1. Data Resources

This paper used unbalanced panel data from 31 mainland provinces from 2001 to 2016 to empirically analyze the impact of setting up “two-oriented society” pilot zones on environmental improvement and economic growth. The experimental area of the “two-oriented society” was approved in December 2007, so 2008 was taken as the beginning node of the observation period after the policy took effect. After 2017, the environmental regulation policy—the “Air Pollution Prevention and Control Action Plan”—became stricter in ten provinces and regions of North China, and coal was replaced by natural gas for winter heating. The effect of limiting atmospheric particulate emissions may be superimposed on the policy effect of the “two-oriented society”, as well as the policy effect from the negotiations with local governments for environmental supervision. Therefore, the policy observation window period of this paper was set to 2016. The data came from the EPS global statistical data platform, the National Bureau of Statistics, the China Economic Network statistical database, the Columbia University Earth Observing System Data and Information System (EOSDIS System), and the average annual PM2.5 data of prefecture-level cities in China, obtained with the help of NASA satellite data. The empirical analysis of this paper aims to simulate the energy conservation, emission reduction, and economic growth of the provinces in the “two-oriented society” pilot zone with the weighted average observation characteristics of other provinces. In this respect, the treatment group consists of Hubei province and Hunan Province, and the control group consists of 29 other provinces.

The study of the environment–economy synergistic improvement effect in this paper involves two aspects: one is economic growth, and the explained variable is the logarithm of per capita GDP of Hubei and Hunan provinces; the other is energy conservation and emission reduction. The explained variables are the log value of the total volume of general industrial solid waste emissions, the log value of the PM2.5 particulate concentration, and the log value of the total volume of industrial sewage emissions in Hubei province and Hunan province. In order to obtain synthetic control results and to calculate them through the optimal combination weights, this paper selected imports and exports, the total volume of retail sales, fixed investments, the general budgetary expenditure of finance, the population of permanent residents, the added value of secondary industry, the foreign technology import contract value, the patent application acceptance number, and private car ownership as the explanatory variables. (See Appendix A Table A1.)

### 3.2. Study Design

This paper took the historical event of the establishment of the “two-oriented society” comprehensive supporting reform pilot zone in 2008 as a case study to evaluate the impact of the establishment of the “two-oriented society” pilot zone on pollutant emissions and economic growth in Hubei and Hunan provinces. This article used the synthetic control method (SCM), which was proposed by Abadie and Gardeazabal and then further improved by Abadie and others [73,74]. The idea of the SCM is to construct the synthetic control object through linear fitting, so as to act as the proxy for provinces that have not set up a “two-oriented society” test area, and then estimate it through counterfactual tools. The policy effect here is the difference between the treated unit and the control unit. The difference-in-differences method (DID) is usually used for evaluation in policy research, and it has certain conditions in use. Firstly, the treated unit and the control unit are comparable before the policy intervention, but this condition is difficult to meet. Secondly, the treated unit itself has subjective selection bias. In order to overcome the shortcomings of the multiple difference method mentioned above and evaluate the policy more accurately, this paper finally analyzed it through the SCM.

Assume that a province exists in the sample, and it establishes the “two-oriented society” pilot zone in time $T_{0}$ ($1\leq T_{0}<T$) and is Province 1. In addition, there are N provinces that have not set up a “two-oriented society” pilot zone. Here, $Y_{1it}$ represents the result of province i setting up the “two-oriented society” pilot zone in period t, $Y_{0it}$ represents the result of province i not setting up the “two-oriented society” pilot zone in period t, and the causal effect is as follows:

(1) $$\tau_{it}=Y_{1it}-Y_{0it},\;i=1,\ldots ,\;N+1,\;t=1,\ldots ,\;T$$

In order to distinguish the differences among provinces, the intervention status of each province i in period t is expressed as $D_{it}$, which indicates whether each province has set up a “two-oriented society” pilot zone in a certain period. $D_{it}=1$ indicates that province i has set up a “two-oriented society” pilot zone in period t, and the value for provinces that have not been set up is 0. Thus, the following formula can be obtained:

(2) $$Y_{it}=D_{it}Y_{1it}+(1-D_{it})Y_{0it}=Y_{0it}+\tau_{it}D_{it}$$

When the province is subject to the establishment of the “two-oriented society” pilot area of this policy intervention, for $t>T_{0}$, at this time,

(3) $$D_{1t}=1,\tau_{1t}=Y_{11t}-Y_{01t}=Y_{1t}-Y_{01t}$$

When $t>T_{0}$, the effect of the policy intervention in the first province can be found as $Y_{11t}$, but the focus of policy evaluation lies in the counterfactual results $Y_{01t}$ after the period $T_{0}$ and how to observe the characteristic values of Province 1 more accurately. Province 1’s counterfactual results $Y_{01t}$ can be assessed by using the following model:

(4) $$Y_{0it}=\delta_{t}+\theta_{t}Z_{i}+\lambda_{t}\mu_{i}+\varepsilon_{it},\;i=1,\ldots ,\;N+1,\;t=1,\ldots ,\;T$$

Among them, the time fixed effect $\delta_{t}$ has the same influence in all provinces, and $Z_{i}$ is an observable control variable, which is not affected by disposal and time changes. Here, the unknown coefficient vector of 1×K dimension is $\theta_{t}$, the common factor of the 1∗ F dimension that can be obtained through observation is $\lambda_{t}$, the F×1 dimension coefficient vector is $\mu_{i}$, and the impact that cannot be observed is $\varepsilon_{it}$.

In order to evaluate the periods before and after the establishment of the “two-oriented society” pilot zone, the counterfactual result is $Y_{01t}$. The constructed vector $W=(\omega_{2},\ldots ,\omega_{N+1})$ is the N∗1 dimension, which satisfies the condition $\omega_{j}\geq 0,j=2,\ldots ,N+1$. The value range of the weight is positive, and the sum of the weights is 1, so as to avoid the formation of deviation. In the control group, the weighted average of all provinces is the weight vector, and the model settings are as follows:

(5) $$\sum_{j=2}^{N+1}\omega_{j}Y_{jt}=\delta_{t}+\theta_{t}\sum_{j=2}^{N+1}\omega_{j}Z_{j}+\lambda t\sum_{j=2}^{N+1}\omega_{j}\mu_{j}+\sum_{j=2}^{N+1}\varepsilon_{jt}$$

As for the weight vector, we suppose that $W^{*}=(\omega_{2}^{*},\ldots \omega_{N+1}^{*})$, so

(6) $$\sum_{j=2}^{N+1}\omega_{j}{}^{*}Y_{j1}=Y_{11}\sum_{j=2}^{N+1}\omega_{j}{}^{*}Y_{j2}=Y_{12},\ldots ,\sum_{j=2}^{N+1}\omega_{j}{}^{*}Y_{jT_{0}}=Y_{1T_{0}}\sum_{j=2}^{N+1}\omega_{j}{}^{*}Z_{j}$$

Existing research suggests that if $\sum_{t=1}^{T_{0}}\lambda_{t}^{\prime }\lambda_{t}$ is nonsingular, then

(7) $$Y_{01t}-\sum_{j=2}^{N+1}\omega_{j}{}^{*}Y_{j1}=\sum_{j=2}^{N+1}\omega_{j}{}^{*}\sum_{s=1}^{T_{0}}\lambda_{t}\left[\sum_{n=1}^{T_{0}}\lambda^{n}\sum_{s=1}^{T_{0}}\lambda_{t}\right]\lambda^{s}(\varepsilon_{js}-\varepsilon_{1s}-\sum_{j=2}^{N+1}\omega_{j}{}^{*}(\varepsilon_{jt}-\varepsilon_{1t})$$

If a long period of time passes before the establishment of the “two-oriented society” pilot zone, the above formula will approach 0, and the policy effect of the impact of the establishment of the “two-oriented society” pilot zone in Province 1 can be obtained, namely

(8) $${\hat{\tau }}_{1t}=Y_{1t}-\sum_{j=2}^{N+1}\omega_{j}{}^{*}Y_{jt}t=T_{0}+1,\ldots ,T$$

Here, $\sum_{j=2}^{N+1}\omega_{j}{}^{*}Y_{jt}$ can be regarded as the unbiased estimator of $Y_{01t}$. The evaluation of the “two-oriented society” pilot zone can be obtained from the derivation of the above model, and finding the weight vector $W^{*}$ is the key problem to analyze the impact effect.

According to Abadie et al. [73], the distance function is adopted to minimize the distance between $X_{1}$ and $X_{0}W$, and $W^{*}$ is the solution of

(9) $$\min\|X_{1}-X_{0}W\|=\sqrt{\left(X_{1}-X_{0}W)V(X_{1}-X_{0}W\right)}$$

V is the symmetric positive definite matrix M∗M. The Synth program package developed by Abadie was used to obtain the optimal weight vector $W^{*}$, and the control group synthesis of the synthetic control method can be realized [74].

## 4. Results

### 4.1. Reduction Effects of Pollutants

In order to study the reduction effect of ECER in the “two-oriented society” pilot area, we focused on the impact on solid, gas, and liquid. In this paper, the total emissions of general industrial solid waste, the PM2.5 particulate concentration, and the total emissions of industrial wastewater were taken as the policy evaluation variables, respectively, and the difference between the treatment group and the control group was compared to analyze the reduction effect after the establishment of the “two-oriented society” pilot area.

#### 4.1.1. PM2.5 Particulate Matter Concentration

The control group was weighted and a new synthetic treatment group was generated by the SCM. The control group consists of Hubei province and Hunan province, and the variable of the evaluation policy is the PM2.5 particulate density. The synthetic PM2.5 of Hubei province can be seen in Table 1. The results show that Jilin province accounts for the largest proportion, and the weight of Jilin province is 38.8%. Among the provinces that comprise Hunan, Guangxi has the largest proportion, with a weight of 47.5%. To avoid linear interpolation problems, we can change the weight of composite provinces by changing the target province. In this paper, the root-prediction-mean-squared error (RMSPE) of the SCM was analyzed; the value for Hubei province is 0.05252, and the value for Hunan province is 0.05267, indicating that the imitative effect is accurate.

**Figure 1:**
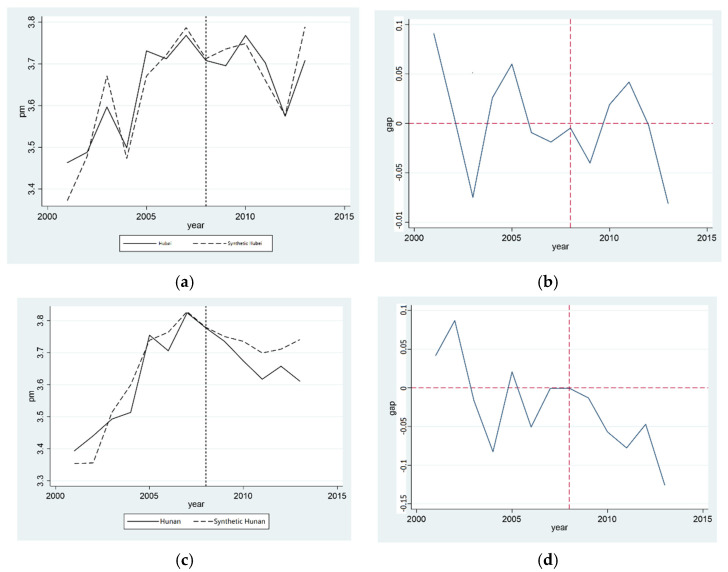
Comparison of PM2.5 particulate concentration reduction effect in provinces established in the “two-oriented society” pilot. (**a**) Comparison of PM2.5 reduction in Hubei; (**b**) Gap in PM2.5 reduction in Hubei; (**c**) Comparison of PM2.5 reduction in Hunan; (**d**) Gap in PM2.5 reduction in Hunan.

Figure 1a,c show the synthetic graphs of the concentration of synthetic PM2.5 particles in Hubei province and Hunan province, respectively, where the solid line represents the actual value of the provinces and the dotted line represents the composite value of the control group. It can be seen that, before the establishment of the pilot zone of the “two-oriented society”, Hunan province had a better fitting effect. This can essentially reflect the concentration of PM2.5 particles in the corresponding provinces of the control group and then be used to reduce the concentration of PM2.5 particles in Hunan province. However, the actual and synthetic values of the PM2.5 concentration in Hubei province have been fluctuating since the establishment of the “two-oriented society” pilot zone, indicating that the establishment of the “two-oriented society” pilot zone has not reduced the PM2.5 concentration in Hubei province, and the effect is not significant.

In order to clearly highlight the influence of the establishment of the “two-oriented society” pilot zone on the PM2.5 concentration, we next calculated the difference between the actual PM2.5 concentration and synthetic PM2.5 concentration in Hubei province and Hunan province, respectively. Figure 1d illustrates the difference for Hunan province. It can be seen that, before 2008, the difference was in a state of fluctuation. After 2008, the difference significantly increased, which was roughly consistent with the change trend of the real PM2.5 particulate concentration in Hunan province in the synthetic figure, and it had a positive promotional effect on the reduction of the PM2.5 particulate concentration in Hunan province. As shown in Figure 1b, the actual and synthetic values of the PM2.5 particulate matter concentration in Hubei province fluctuated before and after the establishment of the “two-oriented society” experimental zone. The establishment of the “two-oriented society” pilot zone did not reduce the PM2.5 particulate matter concentration in Hubei province, and the effect was not obvious. Hypothesis 1a is confirmed in Hunan province, but not in Hubei province; the pilot zone of the “two-oriented society” has some subtractive effects in air pollution control.

#### 4.1.2. Total Emissions of Industrial Wastewater

The control group was weighted and a new synthetic treatment group was generated by the SCM. As before, the control group comprises Hubei province and Hunan province, and the variable of the evaluation policy is the total volume of industrial wastewater emissions. The total volume of industrial wastewater emissions in Hubei province can be seen in Table 2. The results reveal that Sichuan province accounts for the largest proportion, with a weight of 57.7%. Moreover, Sichuan province has a very high weight of 74.8% among the provinces that comprise Hunan province. At the same time, using the SCM of the MSPE to judge the imitative effect, it can be seen that Hubei province has a value of 0.072, proving that the synthetic effect is good, according to the weight of Hubei province’s synthetic research on the “two-oriented society” pilot zone on the total emissions of industrial wastewater in Hubei province, thereby improving the credibility. However, the root-prediction-mean-squared error (RMSPE) of Hunan province reaches 0.17, indicating that the imitative effect is not good and the credibility is reduced.

**Figure 2:**
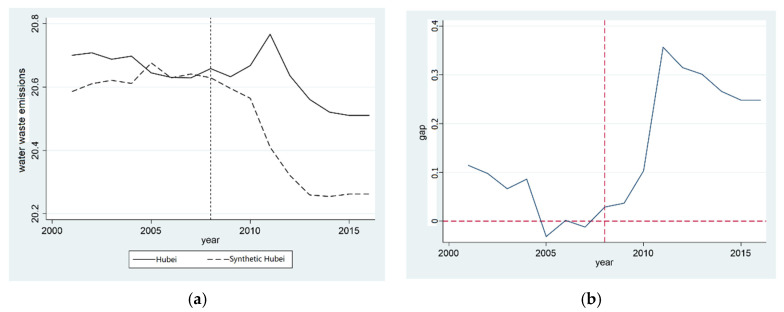

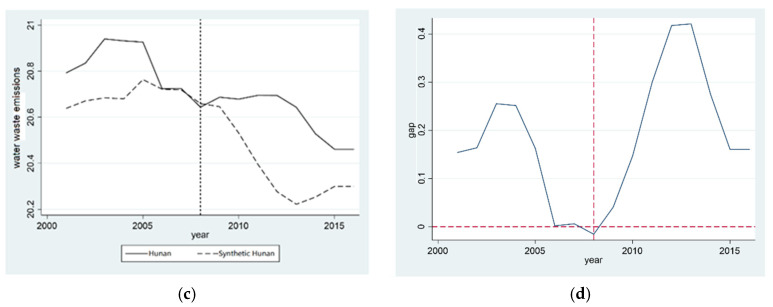
Comparison of total reduction effect of industrial wastewater emissions in provinces established in the “two-oriented society” pilot zone. (**a**) Comparison of wastewater reduction in Hubei; (**b**) Gap in wastewater in Hubei; (**c**) Comparison of wastewater reduction in Hunan; (**d**) Gap in wastewater in Hunan.

Figure 2a,c show the synthetic graphs of the total volume of synthetic industrial wastewater emissions in Hubei province and Hunan province, respectively, where the solid line represents the actual value of the province and the dotted line represents the synthetic value of the control group. The results illustrate that Hubei province had a good fitting effect before the establishment of the “two-oriented society” pilot zone. This shows that the fitted provinces can essentially reflect the total volume of industrial wastewater emissions via the corresponding provinces in the control group. After setting up the “two-oriented society” pilot zone, Hubei province did not have a positive role in reducing the total volume of industrial wastewater emissions, but increased its emissions. Before the establishment of the “two-oriented society” pilot zone, the gap between the actual value and the synthetic value was large, which proves that the establishment of the “two-oriented society” pilot zone did not reduce the total volume of industrial wastewater emissions in Hubei province, and the effect was not significant.

In order to illustrate the impact of the establishment of the “two-oriented society” pilot zone on the total volume of industrial wastewater emissions more clearly, we calculated the difference between the total volume of real industrial wastewater emissions and the total volume of synthetic industrial wastewater emissions, respectively. This gap is in areas where the combined results were significant in Hubei and Hunan provinces. Figure 2b shows the gap for Hubei province. It can be seen that, before 2008, the gap fluctuated between −0.3 and 1.2. After 2008, the gap increased significantly, which was roughly consistent with the change trend of the real total volume of industrial wastewater emissions in Hubei province in the synthetic figure, resulting in the growth of the total volume of industrial wastewater emissions in Hubei province. The total volume of industrial wastewater emissions in Hunan province fluctuated before and after the establishment of the “two-oriented society” pilot zone, as shown in Figure 2d, indicating that the establishment of the “two-oriented society” pilot zone did not reduce the total volume of industrial wastewater emissions in Hunan province, and the effect was not noticeable. Hypothesis 1b has not been verified, and the water pollution treatment effect of the “two-oriented society” pilot zone has not been effectively verified.

#### 4.1.3. Total Emissions of General Industrial Solid Waste

The control group was synthesized by weighting, and a new synthetic treatment group was generated by the SCM. The control group comprised Hubei province and Hunan province, and the variable of the evaluation policy was the total volume of general industrial solid waste emissions. The total emissions of general industrial solid waste in Hubei province can be seen in Table 3. The results show that Henan province accounts for the largest proportion, with a weight of 34.5%. Similarly, among the provinces that make up Hunan province, Henan province accounts for the largest proportion, with a weighting of 39.7%.

**Figure 3:**
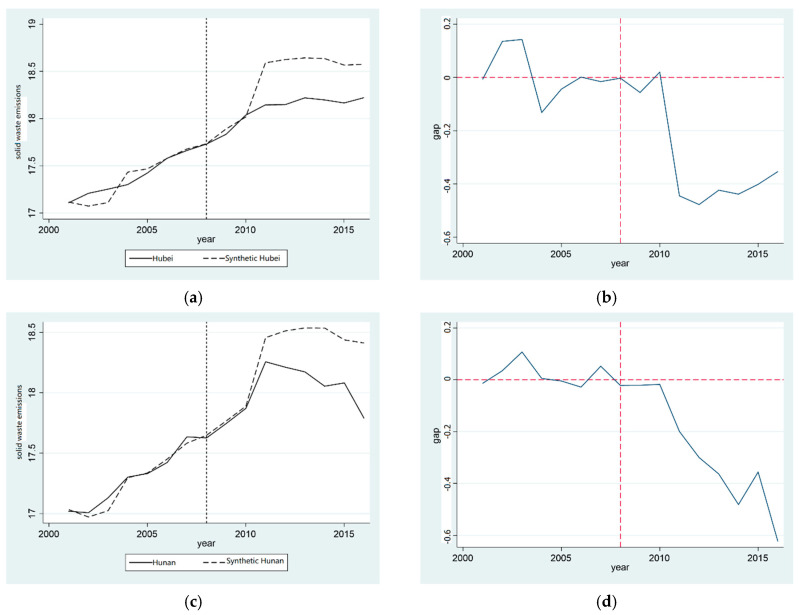
Comparison of reduction effect of total emissions of general industrial solid waste in provinces established in the pilot zone of “two-oriented society”. (**a**) Comparison of solid reduction in Hubei; (**b**) Gap in solid reduction in Hubei; (**c**) Comparison of solid reduction in Hunan; (**d**) Gap in solid reduction in Hunan.

It can be seen from Figure 3a,c that the variation trends of the total emissions of general industrial solid waste in Hubei province and Hunan province are essentially the same, which proves that the actual value fits well with the synthetic value. After the pilot zone to set up the “two-oriented society”, Hubei and Hunan provinces’ general industrial solid waste emissions gradually developed a difference between the actual value and the synthetic value, and this gradually expanded. Moreover, the actual values were lower than the synthetic values, indicating that the establishment of the “two-oriented society” pilot zone can reduce the general industrial solid waste emissions in Hubei and Hunan provinces.

Figure 3b illustrates more intuitively the effect on the general industrial solid waste emissions of setting up the “two-oriented society” pilot zone in Hubei province, revealing the reduction effect of ECER in the “two-oriented society” pilot zone and demonstrating the treatment effect of the “two-oriented society” pilot zone on the total emissions of general industrial solid waste in Hubei province—that is, the difference between “real Hubei province” and “synthetic Hubei province” in the total volume of general industrial solid waste emissions. From 2001 to 2007, the gap fluctuated slightly between 0 and 1.5 and increased rapidly from 2010 to 2011. From 2012 to 2016, the separation effect stabilized. Similarly, it can be seen from Figure 3d that, before and after the establishment of the “two-oriented society” pilot zone, the difference between the actual and synthetic Hunan province in the total volume of general industrial solid waste emissions was between −0.2 and 1, and the difference between the actual value and the synthetic value continued to increase after 2008, indicating the establishment of the “two-oriented society” pilot zone. It can effectively reduce the total emissions of general industrial solid waste in Hunan province. Hypothesis 1c was verified in both test provinces.

### 4.2. Effects of Economic Growth

To study the economic growth effect of setting up the “two-oriented society” pilot zone, this paper captured the difference between the treatment group and the control group, and the GDP per capita was taken as the policy evaluation variable to analyze the influence effect of setting up the “two-oriented society” pilot zone.

The control group was synthesized by weighting through the SCM to generate a new synthetic treatment group. At this point, the control group still consisted of Hubei province and Hunan province, and the variable of the evaluation policy is the GDP per capita. The GDP per capita of synthetic Hubei province can be seen in Table 4. The results indicate that Sichuan province accounts for the largest proportion, with a weight of 53.2%. The GDP per capita of Hunan province can be observed in Table 1. It can be seen that Henan province accounts for the largest proportion, with a weight of 36.8%. The combination formed by construction has a unique solution, indicating that there is no collinearity between the weights of these provinces. To avoid linear interpolation problems, altering the weight of the synthetic province by changing the target province is a suitable method. Generally, in the robustness test of the SCM, the difference in the MSPE ratio of the fitting-mean-squared error before and after the policy occurrence point can better represent the size of the policy effect between the real sample and the robust sample. The MSPE is usually used to accurately observe the difference degree of fitting between established and unestablished provinces. The MSPE comparison results in a poor approximation degree of the model to this province. In this paper, the mean-squared error of prediction when using the SCM is 0.005 in Hubei province and 0.015 in Hunan province, which demonstrates that the synthetic effect is good.

The “two-oriented society” pilot zone was set up in 2008, and the actual value and the synthetic value of GDP per capita between Hubei province and Hunan province began to show differences. The actual value is larger than the synthetic value; after a long period, the results show that the establishment of the “two-oriented society” pilot zone can promote the growth of Hubei and Hunan provinces’ GDP per capita.

**Figure 4:**
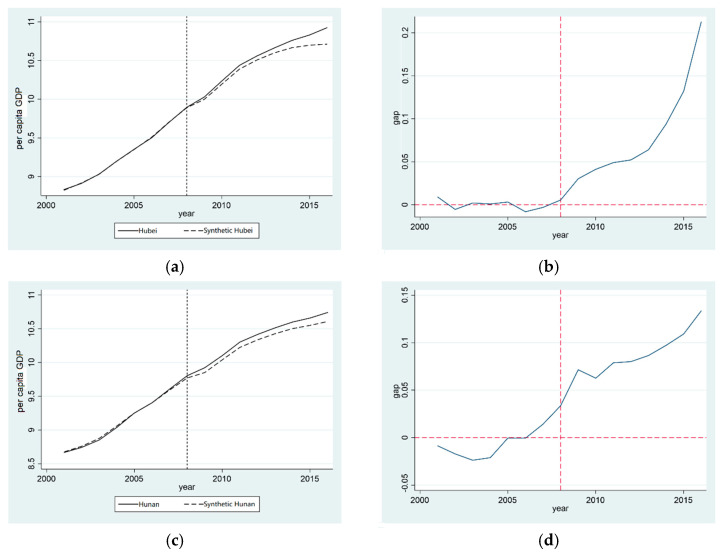
Comparison of GDP per capita reduction effect in provinces established in the “two-oriented society” pilot zone. (**a**) Policy effect of GDP per capita in Hubei; (**b**) Gap in GDP per capita in Hubei; (**c**) Policy effect of GDP per capita in Hunan; (**d**) Gap in GDP per capita in Hunan.

This paper compared the correlation difference between the real Hubei and synthetic Hubei province, as well as the real Hunan province and synthetic Hunan province, before and after the establishment of the “two-oriented society” pilot zone as the treatment effect of the establishment of the “two-oriented society” pilot zone to measure the effect of its establishment on the GDP per capita of Hubei province and Hunan province. Figure 4b,d demonstrate that, from 2001 to 2008, the gap between the real and synthetic GDP per capita of Hubei province remained between −0.1 and 0.1. After the establishment of the “two-oriented society” pilot zone, the gap between them gradually increased, and the SCM shows that the establishment of the “two-oriented society” pilot zone had a positive effect on the growth of the GDP per capita in Hubei province. Similarly, before and after the establishment of the “two-oriented society” pilot zone, the difference between the real and synthetic Hunan GDP per capita essentially fluctuated between −0.2 and 0.3, and the difference between the real and synthetic values was positive after 2008 and continued to increase. The establishment of the “two-oriented society” pilot zone had a positive effect on the growth of the GDP per capita in Hunan province.

Based on the above analysis, it can be seen that Hypothesis 2 is correct: the “two-oriented society” pilot zone promotes the economic growth of Hubei province and Hunan province. The “two-oriented society” pilot zone does not protect the environment by sacrificing growth.

We next aimed to explore whether the establishment of the “two-oriented society” pilot zone or the implementation of other policies caused the difference between the actual value and the synthetic value of the reduction effect of ECER and the economic growth effect in Hubei province and Hunan province. After the sensitivity analysis for the robustness test, to judge whether there is a significant difference, the construction of the synthetic province model needs to be evaluated several times. During the iteration, the method is to delete a sample of the synthetic province model with significant weight and then repeat this until the last province in the weight reorganization is obtained.

**Figure 5:**
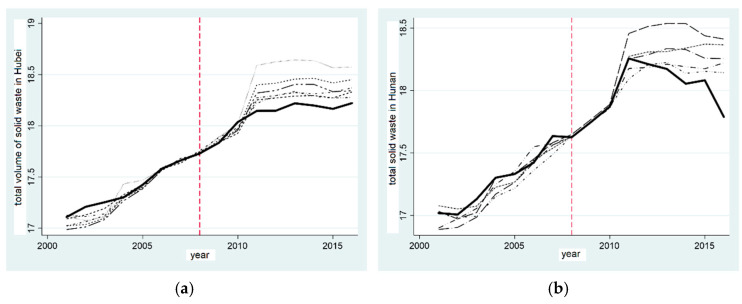
Sensitivity analysis of the total volume of general industrial solid waste emissions in the provinces established in the pilot zone of “two-oriented society”. (**a**) Hubei; (**b**) Hunan.

**Figure 6:**
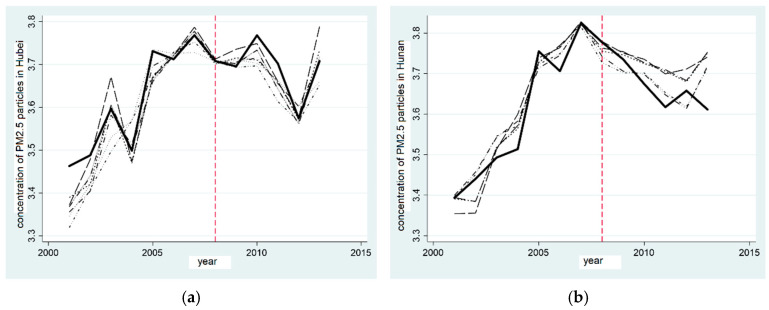
Sensitivity analysis of PM2.5 density in provinces in the “two-oriented society” pilot zone. (**a**) Hubei; (**b**) Hunan.

Figure 5a,b report the robustness test results of the iterative method for setting up the “two-oriented society” pilot zone, excluding Tibet, where large amounts of data were missing. As can be seen from Figure 6, when individuals with weights larger than 0 in the control group were removed one by one, the reduction effect of the “two-oriented society” pilot zone on the total emissions of general industrial solid waste was still positive, thus verifying its robustness.

The results in Figure 6a,b show that the construction of the synthetic Hubei province model requires multiple evaluations, and when iterating, the method deletes a province with significant weight in the synthetic Hubei province model, continuing until the last province in the weight reorganization is obtained. Through the robustness test of the sensitivity analysis, the empirical results demonstrate that Hunan province does not change with the change in the control province, which proves that the policy is effective. Nevertheless, it has no noticeable positive promotional effect on reducing the PM2.5 concentration in Hubei province.

**Figure 7:**
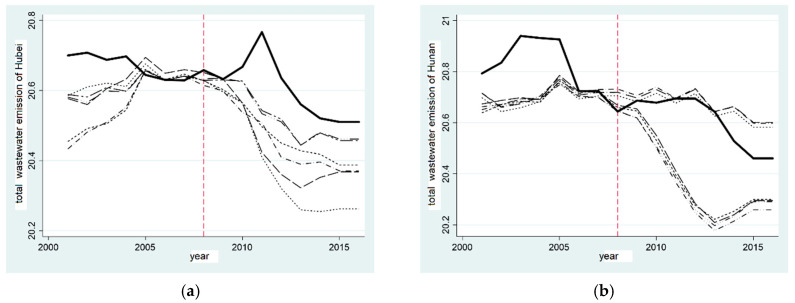
Sensitivity analysis of the total industrial wastewater emissions by provinces in the pilot zone of “two-oriented society”. (**a**) Hubei; (**b**) Hunan.

Further robustness tests were carried out to examine whether there is subjective bias of control group selection that might affect the analysis results, and the sensitivity analysis of the original weight estimation group of the SCM was conducted by the sample iteration method, which proved that the results were still robust. The results are listed in Figure 7a,b. It can be found that the weight composition of the control group is successively changed for Hubei province and Hunan province. The results show that the establishment of the “two-oriented society” pilot zone does not reduce the total volume of industrial wastewater emissions in Hubei province, and the effect was not significant in Hunan province.

**Figure 8:**
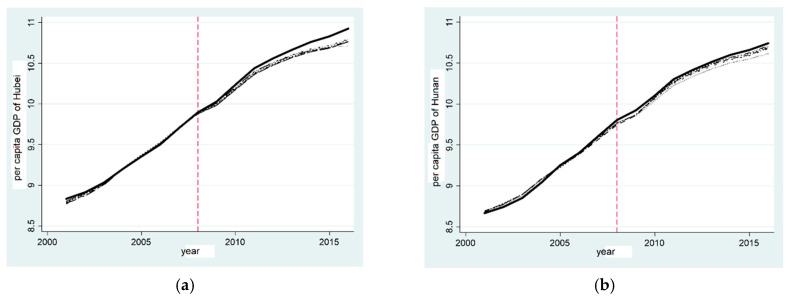
Sensitivity analysis of average GDP of provinces in the “two-oriented society” pilot zone. (**a**) Hubei; (**b**) Hunan.

As shown in Figure 8a,b, the robustness tests of the GDP per capita of Hubei province and Hunan province were conducted by using sensitivity analysis. Taking Hubei province as an example, the construction of the synthetic model of Hubei province needed to be evaluated several times. When iterating, the method deletes a province with significant weight in the synthetic model of Hubei province. Similarly, there are six iterations until the last province in the weight reorganization. Among them, the weight of Hubei province and the weights of other provinces are as follows: Sichuan province, 0.532; Jilin province, 0.195; Liaoning province, 0.194; Henan province, 0.067; Fujian province, 0.012. The sensitivity analysis shows that the control group does not cause a difference.

## 5. Discussion

The results of the above counterfactual analysis indicate that the policy pilot of the “two-oriented society” pilot zone is successful in promoting the development path of regional economic growth and environmental inclusion. According to the analysis results of the three representative pollutants’ reduction in Section 3.1, pollutant reduction levels in the pilot regions have been improved to varying degrees, being significantly different from those in non-pilot provinces. At the same time, as shown in Section 3.2, Hubei and Hunan provinces do not lose economic vitality, and the growth of the GDP was significantly better than that in other provinces in the control group.

However, could this economic effect be the result of other policy interventions over the same period? China is in the process of continuous institutional changes with the reform and opening up, and a number of institutional innovations are constantly promoting economic and social development by means of policy application and practice. First, the study identified whether this policy effect is influenced by the environmental legislation. China’s Environmental Protection Law was enacted in 1989 and was most recently revised in 2014, and the law is in effect nationwide—not only in Hubei and Hunan provinces. Therefore, it can be considered that the conclusion of this paper is not affected by the environmental legislation.

In addition, during the 11th Five-Year Plan period, China’s environmental protection goals were incorporated into the official accountability system, forming a strict set of policy goals, which may also affect local energy conservation and emission reduction efforts [60]. However, environmental accountability is also being implemented across the country—not only in the pilot provinces. Moreover, the implementation of this system began in 2006, which is inconsistent with the time node of 2008 analyzed in this paper. Figure 2 and Figure 3, displaying the results of the SCM, illustrate the difference in the time point of the policy effect. Therefore, environmental accountability can be excluded from influencing the empirical results of this paper.

Finally, we considered whether the policy of promoting economic growth has a similar economic promotion effect on the experimental provinces. Through identifying the major policy pilot projects in the same period, such as the rise of six central provinces, the Yangtze River Economic Belt, the national hygienic civilization city, the national central city, and the free trade zone, it can be clarified that none of the above policy pilot projects can guarantee the policy dividends of Hubei and Hunan provinces or solve the policy spillover problem. In addition, the implementation of some policies is not jointly piloted by the two provinces, such as the construction of the free trade zone, which is contrary to the conclusion of the economic growth effect in this paper. Lastly, the occurrence time of the above policy is inconsistent with the time setting given in Figure 2 in Section 3.2 of this paper. Therefore, we can state that the economic growth effect obtained in the research is not due to the interference of other policy factors.

The pilot construction of the “two-oriented society” pilot zone in China has explored a new path of cleaner production for economic and social development and has provided exciting experimental results regarding the choice of the economic growth path in developing countries. Whether it can lead to a long-term experience of sustainable growth needs further observation.

## 6. Conclusions

Based on the provincial panel data of 31 provinces in China, this paper evaluated the comprehensive economic and social development and environmental emission changes of the pilot provinces of the “two-oriented society” pilot zone, namely Hubei province and Hunan province, from 2001 to 2016. It revealed the remarkable achievements of the “two-oriented society” pilot zone in the fields of economic growth and emission reduction regarding solid, gas, and liquid wastes. The results show that, since the establishment of the “two-oriented society” pilot zone, the GDP per capita of Hubei province has significantly increased, and the total volume of general industrial solid waste has reduced. However, the policy has had no noticeable effect on reducing the concentration of PM2.5 particles and the total volume of industrial wastewater emissions in Hubei province. For the sample of Hunan province, the establishment of the pilot area significantly increased the local GDP per capita, reduced the total volume of general industrial solid waste emissions, and reduced the density of PM2.5 particles, but did not significantly reduce the total volume of industrial wastewater emissions.

The trials on the “two-oriented society” pilot zone suggest that a balanced approach of economic growth and environmental governance could be reached gradually over the course of development driven by institutional innovation, with a view toward achieving such balanced development in the form of incremental economic growth and significant pollution control. Therefore, this paper puts forward the following policy recommendations.

First, market mechanisms should be better used to promote economic growth and reduce pollutant emissions in the pilot areas of government-led policies. The treatment of pollutant emissions has a sequence. Water pollution, the most difficult-to-treat form of pollution at present, also requires better solutions. Therefore, it is advisable to adopt market mechanisms to increase the cost of water pollution emissions and strengthen the economic incentive effect of the popularization of sewage treatment technology, so as to promote polluters to strengthen the treatment of sewage emissions and reduce the emissions of wastewater.

Second, the government should strengthen the inter-provincial prevention and control of air pollution, especially inhalable particles such as PM2.5. The formation of haze in China has a marked regional correlation, illustrating the distribution characteristics of the generation and diffusion zone from north to south in winter. In the north of Hunan province, Hubei province is the dominant region in taking over the drifting haze belt from the north to the south in terms of spatial distance. Hubei province is located in the plain of the middle reaches of the Yangtze River. Thereby, only by strengthening the close cooperation between Hubei province and its northern neighbors, such as Henan province and Anhui province, and carrying out cross-regional haze control and fossil fuel replacement can the problem of PM2.5 emissions be gradually solved. Moreover, the AQI index will perform better due to the improvement in PM2.5.

Third, it is necessary to maintain the leading characteristics of the “two-oriented society” pilot zone, expand and strengthen the environmental protection industry, form a characteristic industrial chain, and establish an industrial-scale advantage. Environmental governance offers an advantage brought about by the industry layout and development momentum, so as to not only control the local pollution emissions more efficiently, but also more actively expand the domestic and foreign environmental protection industry development space and become a common leader in environmental technology innovation and application; this would lead to a broader range of environmental management and pollutant treatment conditions to achieve market competitiveness.

The successful experience of constructing the “two-oriented society” comprehensive reform pilot zone in Hubei and Hunan provinces has provided a new path for developing countries and regions to avoid the environmental Kuznets curve (EKC). The attempt at high-quality growth has made it possible for less developed regions to achieve both growth and energy conservation and emission reduction. In order to achieve the Millennium Development Goals proposed by the United Nations and form the “inverted U-shaped” inflection point of the environmental Kuznets curve in advance, the majority of low- and middle-income countries must moderate the peak of pollution emissions through active environmental governance, guide the decay process of total pollutant emissions, and achieve green development.

The research in this paper still has limitations. First, the assessment of the pollutant emission reduction effect did not cover all major pollutants, but only included a counterfactual estimation of typical types of atmospheric pollution, covering liquid and solid pollutants. Second, a general consideration of the green GDP was not adopted, so it is difficult to compare the results of this paper with other similar empirical results. Third, the paper focuses on the implementation of the “two-oriented society” pilot on the policy effect of pollutant emissions and economic growth as a causal inference; the mechanism analysis was only discussed from a theoretical perspective.

## Figures and Tables

**Table 1:** Nonzero weights for samples of synthetic PM2.5 particulate density.

Synthesis of Hubei Province		Synthesis of Hunan Province	
Sample Provinces	Weights	Sample Provincial	Weights
Jilin	0.388	Guangxi	0.475
Henan	0.373	Shandong	0.365
Shanghai	0.07	Guizhou	0.121
Shandong	0.069	Anhui	0.029
Anhui	0.059	Guangdong	0.01
Chongqin	0.042		

**Table 2:** Nonzero weights of samples for synthetic total emissions of industrial wastewater.

Synthesis of Hubei Province		Synthesis of Hunan Province	
Sample Provinces	Weights	Sample Provinces	Weights
Sichuan	0.577	Sichuan	0.748
Zhejiang	0.148	Anhui	0.094
Inner Mongolia	0.102	Chongqin	0.052
Guangxi	0.091	Guangdong	0.049
Beijing	0.076	Ningxia	0.043
Shandong	0.006	Qinghai	0.014

**Table 3:** Nonzero weights of samples for total emissions of synthetic general industrial solid waste.

Synthesis of Hubei Province		Synthesis of Hunan Province	
Sample Provincial Weights		Sample Provincial Weights	
Henan	0.345	Henan	0.397
Hebei	0.207	Sichuan	0.206
Shandong	0.163	Hebei	0.154
Tibet	0.108	Guangxi	0.108
Sichuan	0.089	Tibet	0.104
Guizhou	0.088	Guangdong	0.032

**Table 4:** Nonzero weights of samples for synthetic GDP per capita.

Synthesis of Hubei Province		Synthesis of Hunan Province	
Sample	Provincial Weights	Sample	Provincial Weights
Sichuan	0.532	Henan	0.368
Jilin	0.195	Sichuan	0.255
Liaoning	0.194	Heilongjiang	0.194
Henan	0.067	Guizhou	0.13
Fujian	0.012	Liaoning	0.053

## Data Availability

The data came from the EPS global statistical data platform, National Bureau of Statistics, China Economic Network statistical database, Columbia University Earth Observing System Data and Information System (EOSDIS System), and the average annual PM2.5 data of prefecture-level cities in China with the help of NASA satellite data.

## Appendix A

**Table A1:** Description statistics of the variables.

Variables	Obs	Mean	Std.Dev.	Min	Max
AGDP	464	10.0397	0.7886	8.0007	11.6795
Pm2.5	464	3.261	0.6287	1.5694	4.4066
Solid_ waste	464	17.6062	1.1038	13.5278	19.9375
Water_ waste	464	19.9997	0.9857	17.3573	21.8095
Trade_volume	464	25.6438	1.7943	21.0093	29.6311
Social_retail	464	26.326	1.1833	22.9249	28.8763
Fix_investment	464	26.8121	1.2145	23.6734	29.3048
Finace_expenditure	464	25.6888	1.0422	22.7894	27.9271
Population	464	26.559	0.7636	24.6803	27.7262
Second_industrial	464	26.5932	1.162	23.2497	28.8869
Import_tech	464	18.8497	1.9947	12.1548	22.9967
Patent	464	9.3241	1.6715	4.8203	13.1469
Car_owner_volume	464	4.5454	1.3241	0.7605	7.3464
